# The toxic guardians — multiple toxin-antitoxin systems provide stability, avoid deletions and maintain virulence genes of *Pseudomonas syringae* virulence plasmids

**DOI:** 10.1186/s13100-019-0149-4

**Published:** 2019-01-31

**Authors:** Leire Bardaji, Maite Añorga, Myriam Echeverría, Cayo Ramos, Jesús Murillo

**Affiliations:** 1 Institute for Multidisciplinary Applied Biology, Universidad Pública de Navarra, 31192 Mutilva, Spain; 2 Instituto de Hortofruticultura Subtropical y Mediterránea «La Mayora», Universidad de Málaga-CSIC, Área de Genética, Universidad de Málaga, Campus de Teatinos s/n, 29010 Málaga, Spain

**Keywords:** Postsegregational killing, Native plasmid evolution, IS*801*, Replicative transposition, IS*91* family, Olive knot disease, Pathogenicity, MITEs, One-ended transposition, *Pseudomonas savastanoi*

## Abstract

**Background:**

*Pseudomonas syringae* is a γ-proteobacterium causing economically relevant diseases in practically all cultivated plants. Most isolates of this pathogen contain native plasmids collectively carrying many pathogenicity and virulence genes. However, *P. syringae* is generally an opportunistic pathogen primarily inhabiting environmental reservoirs, which could exert a low selective pressure for virulence plasmids. Additionally, these plasmids usually contain a large proportion of repeated sequences, which could compromise plasmid integrity. Therefore, the identification of plasmid stability determinants and mechanisms to preserve virulence genes is essential to understand the evolution of this pathogen and its adaptability to agroecosystems.

**Results:**

The three virulence plasmids of *P. syringae* pv. savastanoi NCPPB 3335 contain from one to seven functional stability determinants, including three highly active toxin-antitoxin systems (TA) in both pPsv48A and pPsv48C. The TA systems reduced loss frequency of pPsv48A by two orders of magnitude, whereas one of the two replicons of pPsv48C likely confers stable inheritance by itself. Notably, inactivation of the TA systems from pPsv48C exposed the plasmid to high-frequency deletions promoted by mobile genetic elements. Thus, recombination between two copies of MITE*Psy2* caused the deletion of an 8.3 kb fragment, with a frequency of 3.8 ± 0.3 × 10^− 3^. Likewise, one-ended transposition of IS*801* generated plasmids containing deletions of variable size, with a frequency of 5.5 ± 2.1 × 10^− 4^, of which 80% had lost virulence gene *idi*. These deletion derivatives were stably maintained in the population by replication mediated by *repJ*, which is adjacent to IS*801*. IS*801* also promoted deletions in plasmid pPsv48A, either by recombination or one-ended transposition. In all cases, functional TA systems contributed significantly to reduce the occurrence of plasmid deletions in vivo.

**Conclusions:**

Virulence plasmids from *P. syringae* harbour a diverse array of stability determinants with a variable contribution to plasmid persistence. Importantly, we showed that multiple plasmid-borne TA systems have a prominent role in preserving plasmid integrity and ensuring the maintenance of virulence genes in free-living conditions. This strategy is likely widespread amongst native plasmids of *P. syringae* and other bacteria.

**Electronic supplementary material:**

The online version of this article (10.1186/s13100-019-0149-4) contains supplementary material, which is available to authorized users.

## Background

Plasmids are dispensable extrachromosomal elements widely distributed in bacteria, facilitating their survival and the colonization of eukaryotic hosts [1–4]. The plasticity and transmissibility of plasmids contribute to a rapid dissemination of resistance and virulence genes, thus promoting the emergence of uncontrollable bacterial diseases, both in clinical and agricultural settings [5–8]. However, plasmids are usually large and exist in several copies per cell, potentially imposing a significant metabolic burden to the cell, which might facilitate the emergence of plasmid-free derivatives in the absence of selection for plasmid-borne characters [7, 9]. This metabolic cost can be lowered by diverse plasmid-host adaptations, such as deletions, mutations in the plasmid replication machinery, or chromosomal mutations [7, 9]. Additionally, plasmids can increase their stability by conjugal transfer and/or by carrying a battery of specifically dedicated genetic determinants, classified into three main categories [9–11]. Partition determinants, in the first category, direct the active segregation of plasmid molecules during cell division. All low-copy plasmids appear to contain a partition system, which usually consists of an operon of two genes plus a specific DNA sequence for recognition. Multimer resolution systems comprise the second category and include recombinases that resolve plasmid cointegrates and maximize the number of plasmid copies available at cell division. The third category, postsegregational killing systems, include toxin-antitoxin (TA) systems and, less prominently, restriction modification loci; these systems ensure plasmid maintenance by inhibiting cell growth.

The *Pseudomonas syringae* complex is considered the most important bacterial plant pathogen in the world [12]. Most strains contain plasmids with an array of adaptive genes that increase aggressiveness, expand their host range, and confer resistance to antibacterials or to UV light [1, 6, 13–15]. Most of these plasmids belong to the so-called pPT23A-family plasmids (PFP) group, characterized by sharing the highly conserved RepA-PFP replicon. These replicons are highly plastic and adaptable, and strains often contain two or more stably co-existing PFP plasmids [6, 16–18]. Insertion sequences, transposons and miniature inverted-repeat transposable elements (MITEs) can account for at least a third of a PFP plasmid, actively participating in the acquisition and exchange of adaptive characters [17–21]. Insertion sequence IS*801* (1.5 kb), and its isoforms, is particularly significant because of its relatively high transposition frequency, its common association with virulence genes and its ability to undergo one-ended transposition, whereby the element can mobilize adjacent DNA [19, 21, 22]. Additionally, plasmids of *P. syringae* have a mosaic structure and often share extensive regions of similarity, suggesting their evolution through the acquisition and loss of large DNA regions in a multistep process [14–17, 20, 23]. Despite this, plasmid profiles of individual strains appear to be characteristic and stable, although certain plasmids can be lost with high frequency under certain culture conditions [1, 24–27]. Agricultural settings exert a strong selection pressure on *P. syringae* populations, generally towards highly virulent clones adapted to single hosts, which can be accomplished both by gain and loss of certain virulence genes [23, 28]. However, *P. syringae* is an opportunistic pathogen whose life cycle primarily occurs in a variety of outside-host environments, including living on the surface of plants without causing disease [29]. It is not clear what mechanisms are driving the maintenance of virulence genes in free-living populations, where selection pressure for pathogenicity should be predictably low. Although diverse potential stability determinants were identified among PFP plasmids [15–18, 30–32], it is not yet clear whether or not they are functional and what their role in the bacterial life cycle is.

*P. syringae* pv. savastanoi NCPPB 3335 causes tumours in olive (*Olea europaea*) and is a prominent model for the study of the molecular basis of pathogenicity on woody hosts [33, 34]. This strain contains three PFP virulence plasmids pPsv48A (80 kb), pPsv48B (45 kb) and pPsv48C (42 kb) [18]. Plasmid pPsv48A carries the virulence gene *ptz*, involved in the biosynthesis of cytokinins, and the Type III effector gene *hopAF1*; pPsv48B carries the Type III effector gene *hopAO1* and, in turn, plasmid pPsv48C carries the virulence gene *idi*, potentially involved in cytokinin biosynthesis. Both pPsv48A and pPsv48C are essential for the production of tumours in olive plants [18, 35], whereas pPsv48B contributes to fitness and virulence *in planta* [36]. Although pPsv48A and pPsv48B can be cured, pPsv48C is remarkably stable and could not be evicted from strain NCPPB 3335 [18], perhaps because it carries two different replicons [37]. We were interested in the identification and characterization of the stability determinants of the plasmid complement of strain NCPPB 3335, to gain insights into the mechanisms allowing the long-term maintenance of PFP plasmids and the dynamics of virulence genes.

Here, we determined that the three virulence plasmids from *P*. *syringae* pv. savastanoi NCPPB 3335 carry from one to seven functional stability determinants of different types, including three highly active TA systems in both pPsv48A and pPsv48C, although the two replicons in pPsv48C are likely sufficient for full stability. We serendipitously discovered that the mobile genetic elements IS*801* and MITE*Psy2* promote plasmid deletions and reorganizations with very high frequency. These derivatives are, however, efficiently excluded from the bacterial populations thanks to multiple plasmidic TA systems, which simultaneously favour the maintenance of virulence genes *ptz* and *idi* when outside the plant.

## Results

### Identification of putative stability determinants in the three native plasmids

**Fig. 1:**
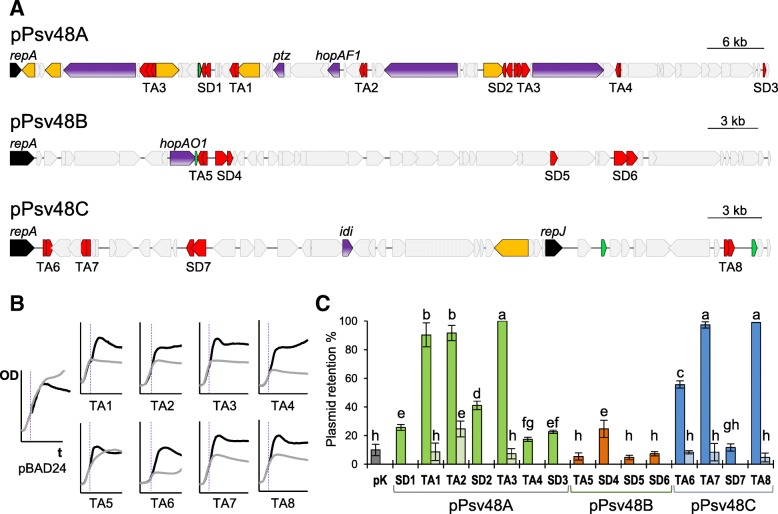
Functional analysis of putative stability determinants from the three native plasmids of *P. syringae* pv. savastanoi NCPPB 3335. **a** Maps of the native plasmids showing the relative position of the stability determinants analysed (red; Table 1), replication initiator protein genes (black), copies of the IS*801* isoform CRR1 (orange), MITEs (green) and virulence genes (purple). **b** Growth patterns of *E. coli* NEB10β containing the toxin gene from the indicated TA systems cloned behind a P_BAD_ promoter, or the empty vector (pBAD24). The vertical dashed line indicates the time when cultures received glucose (black lines), which repressed expression, or arabinose (grey lines), which induced expression. Values of OD_600_ (OD) versus time (t) are the average of three replicates; graphs are representative of at least 4 independent clones. **c** Bars indicate the percentage (mean ± sd) of *P. syringae* pv. syringae B728a cells retaining pKMAG-C alone (pK) or the cloned stability determinants tested in this study (panel **a**; Table 1). For TA systems leading to > 50% of plasmid retention, we show to their right retention values given by their corresponding antitoxins cloned alone. Experiments were repeated three times, each with three replicates. Means with different letters are significantly different (one-way ANOVA and Duncan’s multiple range test; *p* < 0.05)

We identified a total of 15 putative stability determinants, each consisting of one to three coding sequences (CDSs), from the complete sequence of pPsv48A, pPsv48B and pPsv48C (Table 1 and Fig. 1a; see Materials and Methods). These were annotated as four partition systems (SD1, SD4, SD6 and SD7), a multimer resolution system (SD2), a CopG plasmid copy-number regulator (SD3), a plasmid killer protein (SD5), and eight TA systems (TA1 to TA8).

**Table 1:** Putative stability determinants identified in the three native plasmids of *P. syringae* pv. savastanoi NCPPB 3335

Plasmid and determinant^a^	Locus Tag^b^	Deduced product (InterPro family or signature matches)
pPsv48A		
SD1	PSPSV_A0016	Putative partition protein A (IPR027417, IPR025669)
	PSPSV_A0015	Ribbon-helix-helix protein, CopG family (none predicted)
TA1	PSPSV_A0020	Putative addiction module antitoxin, RelB/DinJ family protein (IPR007337)
	PSPSV_A0019	Putative toxin of the YafQ-DinJ toxin-antitoxin system (IPR004386), addiction module toxin, RelE/StbE family (IPR007712)
TA2	PSPSV_A0032	Predicted transcriptional regulator, ribbon-helix-helix protein, CopG (IPR010985)
	PSPSV_A0031	Putative plasmid stabilization system protein; RelE/ParE toxin family (IPR007712)
SD2^c^	PSPSV_A0042	StbA, putative stability/partitioning determinant, resolvase (IPR036162)
	PSPSV_A0041	MvaT-like transcriptional regulator (IPR035616)
TA3	PSPSV_A0043^d^	StbC, Arc-type ribbon-helix-helix protein, putative antitoxin (IPR013321)
	PSPSV_A0044^d^	StbB, putative ribonuclease of the VapC family (IPR022907)
	PSPSV_A0045^d^	StbA, resolvase (IPR036162)
TA4	PSPSV_A0051	Putative RelB/DinJ family addiction module antitoxin (none predicted)
	PSPSV_A0050	Putative RelE/StbE family addiction module toxin (IPR007712)
SD3	PSPSV_A0067	Putative transcriptional regulator; CopG/Arc/MetJ DNA-binding domain-containing protein, possibly responsible for the regulation of plasmid copy number (IPR002145)
pPsv48B		
TA5	PSPSV_B0012	Hypothetical protein, putative plasmid maintenance component (IPR021558, DUF3018)
	PSPSV_B0011	Putative Mazf-like toxin, *ccdB* family (IPR003477)
SD4	PSPSV_B0013	ParA/YafB type stability/partitioning protein, cobyrinic acid ac-diamide synthase (IPR027417)
	PSPSV_B0014	Stability/partitioning protein (none predicted)
SD5	PSPSV_B0038	IncN plasmid killer protein (IPR009989)
SD6	PSPSV_B0042	Putative stability/partitioning determinant (IPR027417)
	PSPSV_B0043	Hypothetical protein (none predicted)
pPsv48C		
TA6	PSPSV_C0003	Putative RelE/StbE family antitoxin, stability determinant (none predicted)
	PSPSV_C0004	Putative RelE/StbE family toxin, stability determinant (IPR007712)
TA7	PSPSV_C0008	Putative CopG family transcriptional regulator (IPR010985; IPR013321)
	PSPSV_C0007	Putative addiction module toxin, plasmid stabilization protein (IPR007712)
SD7^e^	PSPSV_C0017	Putative ParA family protein (IPR027417)
	not annotated	Hypothetical protein (none predicted)
TA8	PSPSV_C0050	Putative antitoxin (none predicted)
	PSPSV_C0051	Putative addiction module toxin, RelE/StbE family (IPR007712)

^a^*TA* toxin-antitoxin system, *SD* generic stability determinant

^b^The listing order indicates direction of transcription

^c^Gene PSPSV_A0042 shows 90% nt identity to gene PSPSV_A0045 in determinant TA3

^d^Genes PSPSV_A0043/44/45 are 100% identical to genes PSPSV_A0007/8/9, respectively

^e^SD7 was cloned containing an unannotated CDS 3′ of PSPSV_C0017 (fragment containing positions 9861–11,121 of FR820587), which could be part of a *par* operon

The deduced products of the putative TA systems had typical protein signatures (Table 1), except the antitoxins of systems TA4, TA6 and TA8. Moreover, the eight respective toxin genes, except that from TA5, lead to cell growth arrest when highly expressed in *E. coli* NEB10β (Fig. 1b). Together, these results indicate that systems TA1-TA8 are indeed toxin-antitoxin systems, although TA5 might be non-functional or *E. coli* NEB10β might be resistant to the TA5 toxin.

### Plasmids pPsv48A, pPsv48B and pPsv48C contain diverse functional stability determinants

The 15 putative stability determinants from plasmids pPsv48A, pPsv48B and pPsv48C were cloned into pKMAG-C, and the stability conferred to the vector was assayed in the plasmidless strain *P. syringae* pv. syringae B728a (Fig. 1c). pKMAG-C is able to replicate in both *E. coli* and pseudomonads [37], and is highly unstable in *P. syringae*.

All seven determinants tested from pPsv48A (Table 1), significantly increased stability of pKMAG-C to varying degrees (Fig. 1c). Four are TA systems, although only three of them conferred very high levels of stability. As expected, these TA systems were functional only when cloned completely, but not when the putative antitoxin was cloned by itself (Fig. 1c), although the antitoxin from system TA2 on its own conferred moderate levels of stability. System TA3 is widespread in pseudomonads e.g. [32, 38, 39] and it is an operon of the TA genes *stbCB* plus the putative resolvase *stbA* (Table 1). Constructs containing either *stbCBA* or only genes *stbCB* conferred equal high levels of stability (not shown); therefore, we evaluated the possible contribution of *stbA* to stability by cloning it separately. *stbA* is the last CDS in the *stbCBA* operon and predictably lacks a promoter; thus, we tested functionality of the *stbA* allele PSPSV_A0042, which is the first CDS of another putative operon (SD2 in Fig. 1) and shows 90% nt identity to the allele in operon *stbCBA*. Operon SD2 also significantly increased stability of pKMAG-C, likely through resolution of plasmid multimers by the StbA resolvase [11], suggesting that operon *stbCBA* might contribute to stability through different mechanisms.

Only one of the four determinants from pPsv48B evaluated here (Table 1) appeared to contribute, albeit modestly, to plasmid stability (Fig. 1c). This was unexpected because low-copy number plasmids usually carry diverse maintenance determinants [40]. The four determinants from pPsv48B showed similar retention values in UPN912 than in strain B728a (not shown), suggesting that lack of activity of three of them (TA5, SD5 and SD6) is not strain-related. Nevertheless, it is possible that pPsv48B contains stability genes that were not included or whose activity was not detected in our assays, and/or that its stability is increased by conjugation [10].

Three TA systems, out of the four determinants tested from pPsv48C, contributed to plasmid stability (Table 1); again, the putative antitoxins did not confer any stability by themselves (Fig. 1c). Remarkably, the eight different TA systems showed distinct behaviours in our assays (Fig. 1c), which varied from no apparent contribution to stability (TA5) to conferring moderate (TA4) to very high stability levels (e.g. TA3 or TA8).

### The two replicons from pPsv48C confer distinct stability

**Fig. 2:**
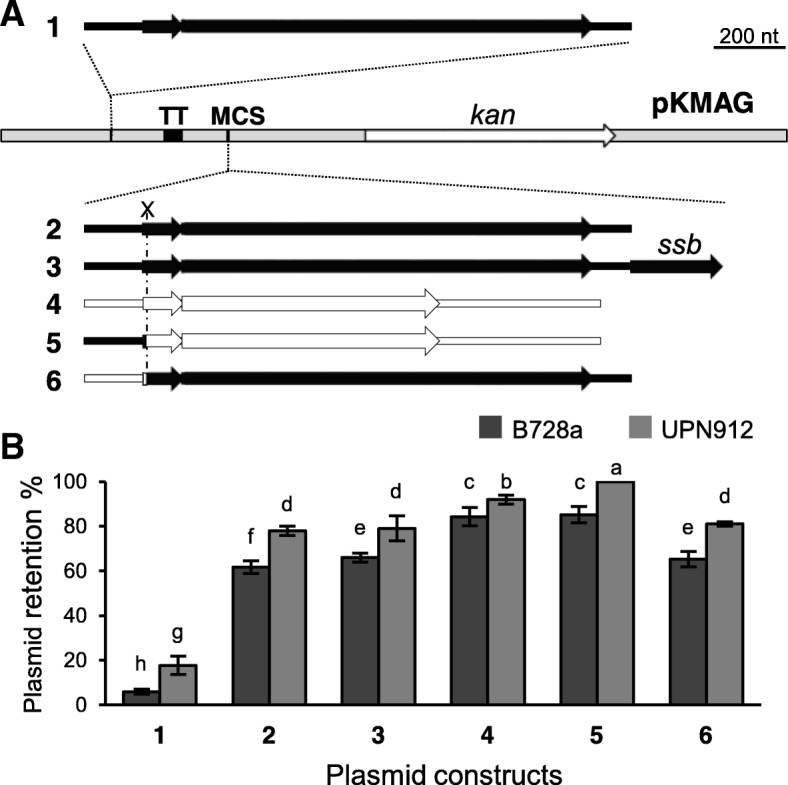
Stability of constructs containing the native RepA-PFP and RepJ replicons from pPsv48C, and their chimeras. **a** Fragments of the RepA-PFP (black) or RepJ (white) replicons, and their chimeras, were cloned at the indicated positions into pKMAG; small and large arrows represent the putative leader peptide and the replication initiator genes, respectively. TT, T4 transcription terminator; MCS, multiple cloning site; *kan*, kanamycin resistance gene. **b** Percentage (mean ± sd) of *P. syringae* pv. syringae B728a cells (dark grey) or of *P. syringae* pv. savastanoi UPN912 cells (light grey) retaining each of the constructs of panel **a** *means* with different letters are significantly different (two-way ANOVA and Duncan’s multiple range test; p < 0.05). Experiments were repeated three times, each with three replicates

To explore the basis of the very high stability of pPsv48C, we evaluated the contribution of the RepA-PFP and RepJ replicons to its maintenance. Therefore, we cloned them into the *E. coli* vector pKMAG and, as before, evaluated stability in the plasmidless strain *P. syringae* pv. syringae B728a (Fig. 2). However, plasmid replicons are often adapted to increase their persistence in their bacterial host e.g. [41, 42]. Therefore, we also tested stability in the plasmidless strain *P. syringae* pv. savastanoi UPN912 (Fig. 2), which derives from the original host strain NCPPB 3335 (Table 2).

**Table 2:** Bacterial strains and plasmids used in this study

Strain/plasmid	Main features^a^	Source or reference
*Escherichia coli*		
NEB10β	Δ(*mrr*-*hsdRMS*-*mcrB*) *deoR recA1 endA1 araD139* Δ(*ara*, *leu*)7697 *galU galK λ*^*−*^ *rpsL nupG*	New England Biolabs
S17–1	Strain used to transfer pDR1 plasmid	[43]
*P. syringae* pv. savastanoi		
NCPPB 3335	Pathotype strain, isolated from a diseased olive tree; contains three native plasmids (pPsv48A, pPsv48B and pPsv48C)	[78]
Psv48ΔAB	NCPPB 3335 derivative, cured of pPsv48A and pPsv48B	[18]
UPN25.1	UPN827 derivative containing pPsv48CΔ25, a 5.5 kb spontaneous deletion derivative of pPsv48C that spans the RepJ replicon, with no TA systems.	[37]
UPN508	Derivative of NCPPB 3335 containing pPsv48A::Tn*5*-GDYN1	[18]
UPN827	Derivative of Psv48ΔAB containing plasmid pPsv48C::Tn*5-*GDYN1	[18]
UPN864	UPN827 derivative, which contains plasmid pPsv48CΔ1	This work
UPN912	Plasmidless derivative of strain NCPPB3335; was obtained by curing pPsv48C::*sacB* from strain UPN1007	[35]
UPN1007	Derivative of Psv48ΔAB containing plasmid pPsv48C::*sacB*	This work
*P. syringae* pv. syringae		
B728a	Plasmidless bean pathogen Cu^R^, Rif^R^, Sm^R^	[79]
Plasmids		
pBlueScript II SK	*E. coli* cloning vector; 2.96 kb, Amp^R^	Stratagene
pBAD24	*E. coli* expression vector, contains a P_BAD_ promoter inducible with arabinose; 4.5 kb, Amp^R^	[76]
pDR1	Delivery vector for Tn*5*-GDYN1, based on pSUP2021; Km^R^, Gm^R^, Sm^R^, Sp^R^; confers sucrose-dependent lethality	[43]
pGEM-T Easy	*E. coli* cloning vector; 3 kb, Amp^R^	Promega
pJET1.2	*E. coli* cloning vector 2.9 kb, Amp^R^	Thermo Fisher Scientific
pK18*mobsacB*	Mobilizable cloning vector, confers sucrose-dependent lethality; Km^R^, Suc^S^	[71]
pKMAG	*E. coli* vector derived from pK184, devoid of the P*lac* promoter and containing the transcriptional terminator and polylinker from pME6041; 2.6 kb, Km^R^; accession no. KX714576	[37]
pKMAG-C	pKMAG containing the minimal RepA-PFP replicon from pPsv48C, replicates in *E. coli* and in *Pseudomonas*; 4.3 kb, Km^R^; accession no. KX714577	[37]
pME6041	Broad host range cloning vector; 5.6 kb, Km^R^	[80]
pPsv48A	Virulence plasmid of NCPPB 3335 (accession n° FR820585); with RepA-PFP replicon; 80.1 kb	[18]
pPsv48A::Tn*5*-GDYN1	Plasmid pPsv48A tagged with Tn*5*-GDYN1 at position 1469	[18]
pPsv48B	Native plasmid of NCPPB 3335 (accession no. FR820586); with RepA-PFP replicon; 45.2 kb	[18]
pPsv48C	Virulence plasmid of NCPPB 3335 (accession no. FR820587); with RepA-PFP and RepJ replicons; 42.1 kb	[18]
pPsv48C::*sacB*	pPsv48C derivative containing the Km^R^-*sacB* cassette from pK18*mobsacB* inserted at position 26,916; Km^R^, Suc^S^	This work
pPsv48C::Tn*5*-GDYN1	pPsv48C containing Tn*5*-GDYN1 inserted at position 37,036; Km^R^, Gm^R^, Suc^S^	This work
pPsv48CΔ1	Derives from pPsv48C::Tn*5*GDYN1 by the spontaneous deletion of 8.3 kb (positions 32,807–41,121 of FR820587) mediated by recombination between two copies of MITE*Psy2*	This work
pPsv48CΔ25	Spontaneous sucrose-resistant deletion derivative from pPsv48C::*sacB*, generated by a one-ended transposition of IS*801*; this plasmid is 5.5 kb long, spanning positions 27,019–32,557 of FR820587	[37]
pRK3A	pRK415 derivative containing genes PSPSV_A0043, PSPSV_A0032 and PSPSV_A0020 cloned in tandem in this order, each with their own promoter and under the control of the P_*lac*_ promoter from the vector; Tc^R^	This work
pRK3C	pRK415 derivative containing genes PSPSV_C0050, PSPSV_C0008 and PSPSV_C0003 cloned in tandem in this order, each with their own promoter and under the control of the P_*lac*_ promoter from the vector; Tc^R^	This work
pRK415	Broad host range cloning vector; 10.5 kb, Tc^R^	[81]

^a^Abbreviations: *Amp* ampicillin, *Cu* copper, *Km* kanamycin, *Gm* gentamicin, *Rif* rifampicin, *Sm* streptomycin, *Sp* spectinomycin, *Suc* sucrose, *Tc* tetracyclin. Superscripts R and S denote resistance or susceptibility, respectively

Construct pKMAG-C, containing the RepA-PFP replicon cloned outside the polylinker of the vector, was highly unstable and was nearly completely lost after only one night of growth (Figs. 1c and 2). This was probably due to a destabilization of the replication control system from an increase in transcription by read-through from the constitutive kanamycin promoter, a phenomenon previously described for replicon RepJ [37]. In fact, its cloning after the transcription terminator of pKMAG significantly increased stability (2 in Fig. 2). Gene *ssb*, which is frequently found downstream of the *repA* gene [17, 18, 31] only showed a marginal contribution to stability (compare 2 and 3, Fig. 2). In turn, the RepJ replicon conferred a significantly higher stability than the RepA-PFP replicon (compare 2 and 4, Fig. 2). Noticeably, all the constructs were significantly more stable in strain UPN912 than in B728a (Fig. 2), suggesting that these replicons are adapted to the bacterial host in which they occur naturally, to maximize their survival.

The RepA-PFP and RepJ replicons consist of two separable functional fragments: a control region, containing the promoter, a putative antisense RNA and a leader peptide, and a replication region, containing the replication initiator protein (*rep*) gene [37]. The approx. 0.3 kb control region determines the transcription rate of the *rep* gene. The RepA-PFP and RepJ replicons share very similar, but not identical control regions preceding the *rep* gene [37], and we hypothesized that this could potentially influence replicon stability. We therefore evaluated the stability of constructs containing chimeric replicons, with the replication control region (Rex-C module) reciprocally swapped [37]. The highest stability in UPN912, but not in strain B728a, was reached with the chimera RepA-PFP:RepJ (control:replication modules; construct 5, Fig. 2), indicating that replicon stability is mostly dependent on the activity of the replication module, but it can be modulated by the control module (Fig. 2).

The significant values of plasmid loss observed for RepJ (Fig. 2) conflicted with the high stability observed for pPsv48C deletion derivatives (not shown), suggesting that we did not clone all the replicon sequences needed for stable replication. We therefore tested the stability of a spontaneous 5.5 kb deletion derivative of pPsv48C (clone pPsv48CΔ25; Table 2), containing the minimal RepJ replicon [37] plus additional DNA that did not include any other potential plasmid maintenance genes. Plasmid pPsv48CΔ25 was maintained in 100% of the cells obtained from starting cultures and after seven sequential culture transfers (1622 and 2804 colonies tested, respectively). In contrast, the RepJ construct in pKMAG (construct 4 in Fig. 2) was retained by 94 ± 2% of UPN912 cells from starting cultures and by only 63 ± 2% of the cells after seven transfers (2366 and 2666 colonies tested, respectively). These results indicate that the native RepJ replicon is larger than the minimal replicon [37] and underscore its high stability in its genetic context.

### A toxin-antitoxin system prevents a deletion in pPsv48C mediated by MITEs

**Fig. 3:**
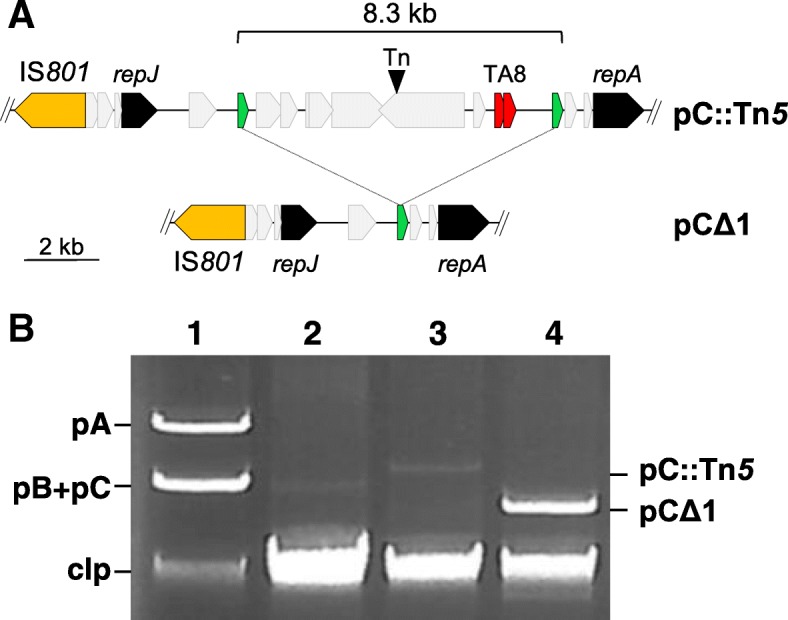
Recombination between two directly repeated copies of MITE*Psy2* causes a deletion on pPsv48C. **a** Partial map of pPsv48C::Tn*5*-GDYN1 (pC::Tn*5*) showing the relative positions of its only copy of the IS*801* isoform, its two replication initiation protein genes (*repJ* and *repA*), and toxin-antitoxin system 8 (TA8). Green block arrows, MITE*Psy2*; inverted black triangle, Tn*5*-GDYN1 (Tn). pCΔ1 is pPsv48CΔ1, containing an 8.3 kb deletion resulting from MITE*Psy2* recombination. **b** Electrophoresed uncut plasmid preparations from: (1) *P. syringae* pv. savastanoi NCPPB 3335; (2) Psv48ΔAB; (3) UPN827, and (4) UPN864. pA, pPsv48A; pB; pPsv48B; pC, pPsv48C; pCΔ1, pPsv48CΔ1; clp, chromosomal DNA and linearized plasmids. Lanes were loaded with equivalent amounts of cell lysates; results are representative of at least 20 independent plasmid preparations

We sought to obtain derivatives of NCPPB 3335 cured of plasmid pPsv48C, and to evaluate the contribution of its three TA systems to stability. We thus constructed strain UPN827, containing a transposon carrying the *sacB* gene (Tn*5*-GDYN1) inserted into pPsv48C (Fig. 3a; Table 2); this allowed us to easily select for plasmid loss by growth in the presence of sucrose [43]. To inactivate functionally the TA systems [44] and facilitate plasmid loss, we constructed pRK3C, containing the three antitoxin genes from pPsv48C cloned in pRK415 (Table 2), and introduced it into UPN827 to neutralise the three corresponding toxins.

We routinely obtained 50 times more sucrose-resistant (suc^R^) colonies with strain UPN827(pRK3C) (38 ± 3 × 10^− 4^ suc^R^ colonies) than with its parental strain UPN827(pRK415) (0.8 ± 0.4 × 10^− 4^ suc^R^ colonies), and this difference was statistically significant. All suc^R^ colonies examined contained an 8.3 kb deletion in pPsv48C caused by the recombination of two direct copies of MITE*Psy2,* as assessed by sequencing, eliminating the *sacB* transposon Tn*5*-GDYN1 (Fig. 3a). One of these plasmids was retained and designated pPsv48CΔ1 (Fig. 3a). These results indicate that, despite its small size (228 nt), MITE*Psy2* is a hot spot for recombination.

In plasmid profile gels of the wild type strain NCPPB 3335, pPsv48C routinely appears with lower intensity than the other two native plasmid bands (Fig. 3b) [18]. Remarkably, bands of plasmid pPsv48CΔ1 were repetitively more intense than those of the wild type plasmid or of pPsv48C::Tn*5*-GDYN1 (Fig. 3b), suggesting that the 8.3 kb deletion caused a higher copy number. We estimated a moderate copy number for plasmids pPsv48A (8.0 ± 1.0), pPsv48B (8.6 ± 1.6) and pPsv48C (6.6 ± 1.2), with no significant differences among them. These are as expected for medium-size native plasmids [45] and similar to the five copies reported for the native plasmid pFKN from *P. syringae* pv. maculicola [20]. Unexpectedly, the estimated copy number of pPsv48CΔ1 (6.9 ± 0.8) was not significantly different from that of pPsv48C. These results indicate that each of the three native plasmids from strain NCPPB 3335 exist in 6–9 copies per cell, and that the 8.3 kb fragment from pPsv48C does not carry any determinant involved in copy number control. This also suggests that structural differences among plasmids could differentially impact their purification by alkaline lysis and questions the use of agarose gel electrophoresis to estimate relative plasmid DNA quantities.

### Toxin-antitoxin systems from pPsv48C prevent accumulation of plasmid deletions mediated by IS*801*

**Fig. 4:**
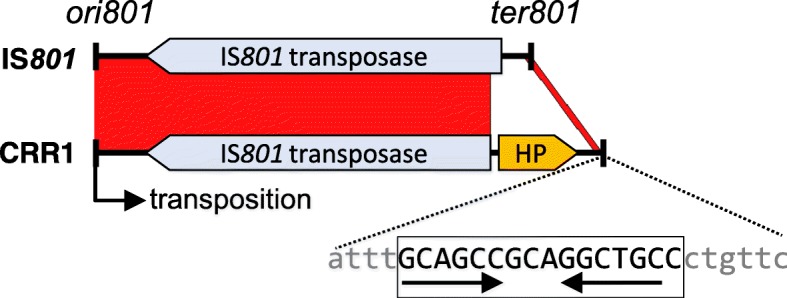
Comparison of the wild type IS*801* with its isoform CRR1. Blastn alignment of IS*801* (X57269; 1512 nt) and CRR1 (from FR820587; 1765 nt); the red bands connecting the two elements indicate collinear regions of identity. CRR1 contains an insertion of 365 nt, causing a deletion of 112 nt that removes the predicted transposase start codon and trims the *ter801* terminus to the endmost 26 nt (expanded sequence). This 26 nt region contains a conserved motif (capital letters) with an inverted repeat sequence (horizontal arrows), probably involved in recognition and interaction with the transposase [46]. HP, hypothetical protein

**Fig. 5:**
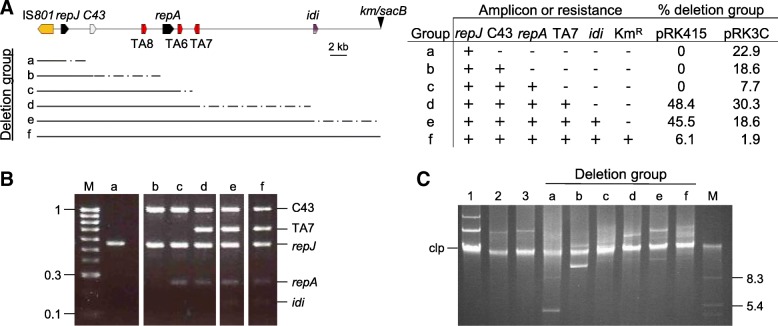
Types of deletions of pPsv48C::*sacB* as influenced by functional toxin-antitoxin systems. **a** Left: Map of pPsv48C::*sacB*; TA6, TA7 and TA8, toxin-antitoxin systems; C43, locus PSPSV_C0043; inverted triangle, Km^R^-*sacB* cassette cloned 0.1 kb 3′ of the IS*801* isoform. Lines under the map indicate the minimum (black line) and maximum (dotted line) extent of DNA transposed by IS*801* on each group of suc^R^ plasmids. Right: Presence (+) or absence (−) of specific amplicons for each of the genes shown, or of resistance (+) and sensitivity (−) to kanamycin. Last two columns indicate the percentage of suc^R^ colonies containing each plasmid group in UPN1007 containing the empty vector pRK415 (310 colonies analysed) or pRK3C, leading to functional inactivation of the TA systems (323 colonies analysed). Gels showing typical patterns of multiplex PCR amplifications (panel **b**) and uncut plasmids (panel **c**) of example clones from each plasmid group. M, molecular weight markers, in kb; clp, chromosomal DNA and linearized plasmids. Lanes: (1) *P. syringae* pv. savastanoi NCPPB 3335; (2) Psv48ΔAB, containing only pPsv48C; and (3) UPN864, containing only pPsv48C::*sacB*

Our preliminary experiments soon indicated that the inactivation of the three TA systems of pPsv48C did not facilitate the isolation of plasmid-cured strains but, instead, led to the recovery of deletion derivatives generated by one-ended transposition of the IS*801* isoform CRR1 (Fig. 4) [18]; for clarity, we will henceforth refer to this isoform as IS*801*. Therefore, strain UPN1007 was used to better estimate the causes and frequency of the different deletions. This strain carries plasmid pPsv48C::*sacB*, containing a Km^R^-*sacB* cassette immediately adjacent to the only IS*801* copy of pPsv48C (Fig. 5); thus, the selection of suc^R^ colonies would allow for the identification and quantification of all types of deletions mediated by one-ended transposition of IS*801*.

The frequency of suc^R^ colonies was 1.8 ± 0.7 × 10^− 4^ for UPN1007 containing the empty vector but significantly higher (5.5 ± 2.1 × 10^− 4^) for strain UPN1007(pRK3C), in which the three TA systems are functionally inactivated (Fig. 5). The plasmid profile and PCR analyses of > 700 independent clones, plus sequencing of 13 of them, indicated that none had lost pPsv48C but showed a plasmid band of ca. 4 to 42 kb resulting from deletions of variable size in this plasmid. All deletion derivatives contained IS*801* and *repJ* (Fig. 5), and sequencing showed that all had a common left border corresponding to the 3′ end of IS*801* (position 27,019 of pPsv48C; Fig. 5a), containing the *ori801* where transposition of this element initiates [46]. The right border of the different plasmid derivatives was GAAC (5 clones) or CAAG (8 clones), which were described as consensus tetramers immediately adjacent to insertions of IS*801* and places for one-ended transposition events to finish [19, 47].

The extent and frequency of deletions generated in pPsv48C, both in UPN1007(pRK415) and in UPN1007(pRK3C), was evaluated in clones growing in SNA by a multiplex PCR analysis (Fig. 5b). Additionally, loss of kanamycin resistance indicated the loss of the Km^R^-*sacB* cassette in the largest deletion derivatives (notice that transpositions ending closer from IS*801* result in the deletion of larger DNA fragments from pPsv48C). The 310 suc^R^ clones examined from strain UPN1007(pRK415) retained plasmids of at least 22 kb, all spanning the three TA operons (TA6–8; Fig. 5a). This was expected because the three TA systems are functional in UPN1007 and their loss would predictably result in growth inhibition. However, around half of the clones had lost gene *idi*, indicating the spontaneous loss of this gene in routine culture conditions with a frequency of 0.9 ± 0.3 × 10^− 4^. The types of deletions were more varied in the 323 suc^R^ clones of UPN1007(pRK3C), containing functionally inactivated TA systems, with nearly half of the clones losing the RepA-PFP replicon and around 80% (4.4 ± 1.9 × 10^− 4^) of them lacking gene *idi* (Fig. 5). Notably, IS*801* was able to transpose the complete length of pPsv48C in both strains (plasmid group f in Fig. 5), although at a low frequency of around 10^− 5^, suggesting that IS*801* is capable of mobilizing more than 40 kb of adjacent DNA. Incidentally, the generation of circular deletion variants of pPsv48C mediated by IS*801* also indicates that, as predicted [47], this element transposes by a rolling circle mechanism.

### Toxin-antitoxin systems also contribute to the maintenance of plasmid pPsv48A and to reducing the occurrence of deletions

**Fig. 6:**
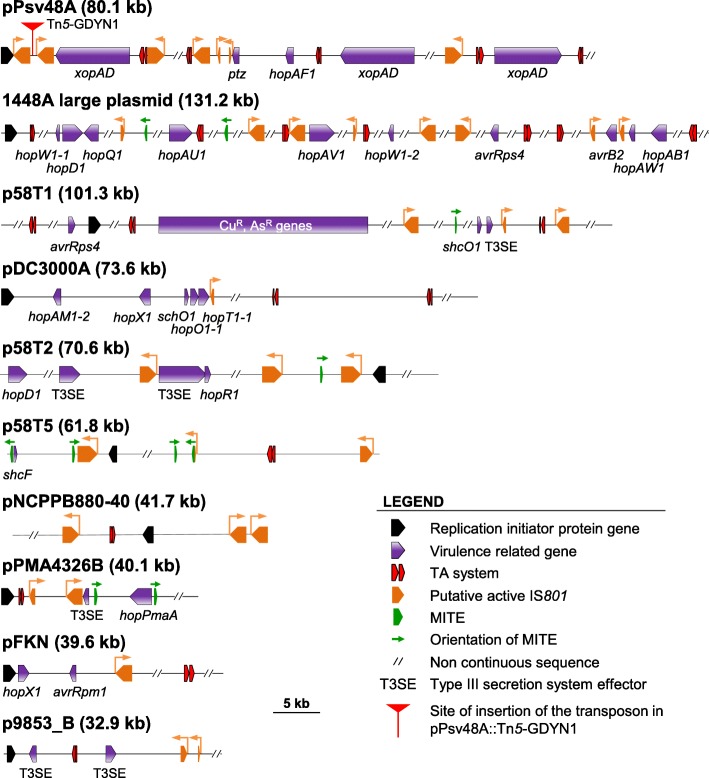
Schematic representation of relevant features found in closed plasmid sequences of *Pseudomonas syringae.* The diagram shows the replication initiator protein genes, virulence genes, TA systems, putative active IS*801* elements and MITEs found in closed plasmid sequences of the *P. syringae* complex. Features are drawn to scale but, for clarity, only pertinent plasmid fragments are shown. The direction of transposition of IS*801* fragments and isoforms is indicated with orange arrows. Harbouring organism and accession numbers for the plasmids are *P. syringae* pv. savastanoi NCPPB 3335, NC_019265 (pPsv48A); *P. syringae* pv. phaseolicola 1448A, NC_007274 (p1448A); *P. syringae* pv. tomato DC3000, NC_004633 (pDC3000A); *P. cerasi* 58^T^, NZ_LT222313 (p58T1), NZ_LT222314 (p58T2), NZ_LT222317 (p58T5); *P. syringae* pv. tomato NCPPB 880, NC_019341 (pNCPPB880–40); *P. cannabina* pv. alisalensis ES4326, NC_005919 (pPMA4326B); *P. syringae* pv. maculicola M6, NC_002759 (pFKN); *P. syringae* pv. actinidiae ICMP 9853, NZ_CP018204 (p9853_B)

Because IS*801* is pervasive in *P. syringae* genomes, we wanted to know if deletions mediated by this element also occurred in other plasmids, and whether or not TA systems are contributing to decrease their frequency. For this, we used strain UPN508, a derivative of strain NCPPB 3335 containing plasmid pPsv48A with an insertion of Tn*5*-GDYN1 located at 1.9 kb 3′ of gene *repA* (Fig. 6) [18]. pPsv48A contains only one replicon and Tn*5*-GDYN1 is inserted between two of the five copies of IS*801* in the plasmid, limiting the types and size of deletions that we can detect, although the experimental setting still allowed us to evaluate the possible occurrence of deletions.

Strain UPN508(pRK415) generated suc^R^ clones with a frequency of 1.1 ± 0.8 × 10^− 4^. From 282 of these suc^R^ clones, plasmid pPsv48A::Tn*5*-GDYN1 was lost in two clones, it contained spontaneous mutations inactivating *sacB* in nine clones, and was reorganized or contained deletions in the remaining ones (Table 3). The majority of the suc^R^ clones, around 90% of the total, contained derivatives of ca. 76 kb; sequencing of three of these clones suggests that they resulted from recombination between the two isoforms of IS*801* flanking the insertion point of Tn*5*-GDYN1 (Table 3), causing its deletion.

**Table 3:** Type and proportion of sucrose-resistant derivatives of pPsv48A::Tn*5*-GDYN1 in the presence or absence of functional toxin-antitoxin systems

	Number (%) of suc^R^ clones^b^			
Plasmid size^a^	UPN508(pRK415)	UPN508(pRK3A)	*ptz* ^c^	Type of event ^d^
- (cured)	2 (0.7)	110 (41.4)	–	
57	1 (0.4)	38 (14.3)	+	Recombination^e^
70	16 (5.7)	4 (1.5)	+	Reorganizations^f^
76	251 (89.0)	111 (41.7)	+	Recombination?^g^
89	9 (3.2)	2 (0.8)	+	Spontaneous mutation in *sacB*
> 90	3 (1.1)	1 (0.4)	+	Reorganizations^h^
Total	282	266		

^a^Approximate size (kb) of the deletion derivative. Total size of pPsv48A::Tn*5*-GDYN1 is around 89 kb

^b^Strain UPN508 contains pPsv48A::Tn*5*-GDYN1, with functional TA systems. The three TA systems of this plasmid are functionally inactivated in the presence of pRK3A, containing the three cloned antitoxins from pPsv48A

^c^Presence (+) or absence (−) of the virulence gene *ptz*, for cytokinin biosynthesis, in the resulting deletion derivatives

^d^All events resulted in deletion of Tn*5*-GDYN1, except the spontaneous mutation in *sacB*

^e^Recombination between IS*801*–1 and IS*801*–4

^f^Diverse group of clones with different, uncharacterized intramolecular reorganizations

^g^The sequence of five clones confirmed that they resulted from recombination between IS*801*–1 and IS*801*–2, but we cannot discard the possibility that some or all of the remaining clones resulted from a transposition of IS*801*–2

^h^These plasmids appeared to result from a transposition of IS*801*–2, terminating precisely at the end of IS*801*–1 as determined by sequencing, that eliminate Tn*5*-GDYN1. According to their relative size on plasmid profile gels, these plasmids must contain an uncharacterized insertion

Functional inactivation of the three TA systems, in strain UPN508(pRK3A), lead to only a modest, but significant increase of the frequency of suc^R^ clones to 3.6 ± 1.5 × 10^− 4^, and to a dramatic change on the plasmid content of these clones (Table 3). The first major difference was that the frequency of loss of pPsv48A was around 1.5 ± 0.2 × 10^− 4^, two orders of magnitude higher than that in UPN508(pRK415) (Table 3). The second major difference was that deletion derivatives of approx. 57 kb, all of which had lost system TA1, appeared around 40 times more frequently than in strain UPN508(pRK415) (Table 3). The frequency of occurrence of the other reorganizations (Table 3) varied no more than four times between both strains. Noticeably, and contrasting with pPsv48C, most of the deletions affecting pPsv48A are likely due to recombination between IS*801* elements instead to one-ended transpositions of IS*801*. This indicates that IS*801* promotes plasmid deletions with high frequency by diverse mechanisms.

### Are multiple toxin-antitoxin systems commonly safeguarding virulence plasmids of *P. syringae*?

Many plasmids of *P. syringae* contain virulence genes and a large amount of mobile genetic elements [1, 2, 6, 17, 18], of which MITEs and IS*801* transpose most frequently [19]. Here we showed that these mobile elements also mediate frequent deletions and reorganizations in two virulence plasmids of *P. syringae* pv. savastanoi NCPPB 3335, and that their carriage of multiple toxin-antitoxin systems allows avoiding these deletions and maintain plasmid integrity. We therefore questioned if this could be a common strategy among virulence plasmids of *P. syringae*.

We found sequences homologous to IS*801* in 53 out of the 78 available closed plasmid sequences from strains of the *P. syringae* group (including *P. cerasi*; December, 2018), with around two thirds of them containing at least one complete or truncated copy of CRR1. This indicates a frequent occurrence of this mobile element in the *P. syringae* pangenome. The sequence of nine of these plasmids, chosen as examples, contained one to eight copies of *ori801* potentially capable of initiating one-ended transposition (Fig. 6); four of them also contained one to four copies of MITE*Psy1*. Likewise, eight of the nine plasmids harboured at least one putative TA system; an extreme case is p1448A-A (131.2 kb), containing eight *ori801* and seven putative TA systems (Fig. 6). These TA systems are also likely limiting the occurrence of deletions, which could potentially eliminate one or more of the virulence genes included in these plasmids (Fig. 6).

## Discussion

Native plasmids of *P. syringae* and other phytopathogenic bacteria often carry genes contributing to virulence and resistance to bactericides, sometimes being essential for pathogenicity [2, 6, 14, 15, 17, 18, 48]. Although they are generally considered moderately to highly stable in the few tested *P. syringae* strains [18, 27], there is a general lack of knowledge of the molecular mechanisms involved in long-term plasmid survival. Here we show that the virulence plasmids from *P. syringae* pv. savastanoi NCPPB 3335 use diverse mechanisms to persist in the cell and maintain their physical integrity.

We identified 11 functional stability determinants among the 15 determinants examined from the three native plasmids of strain NCPPB 3335. These included seven TA systems, two putative partition systems, one putative multimer resolution system and one putative CopG-type copy number control regulator. The four remaining determinants evaluated (TA5, SD5, SD6 and SD7) appeared to be non-functional. It is nevertheless possible that the high instability of the vector used for testing, pKMAG-C, did not allow us to detect their activity as stability determinants, although TA5 is probably non-functional since it did not show activity in *P. syringae* strains B728a and UPN912, and in *E. coli*. We showed that the TA systems are a major stability determinant only for plasmid pPsv48A, increasing its stability by two orders of magnitude. The TA systems do not appear to contribute to the stability of pPsv48C because this plasmid carries two replicons [37] conferring a very high stability level by themselves. In particular, RepJ can be maintained with no apparent plasmid loss for seven sequential culture transfers in the absence of any identifiable maintenance determinants. Notably, these two replicons appear to be adapted to its native host to maximize their stability (Fig. 2). The carriage of several strong stability determinants clearly favours the maintenance of virulence genes but also likely the acquisition of new plasmids and adaptive characters. Virulence genes are frequently found on PFP plasmids [1, 6], which are often exchanged horizontally [2, 49, 50]. This, however, appears to not disturb the previous plasmid complements, because strains of *P. syringae* usually harbour two to six different PFP plasmids [16]. Thus, strong stability determinants likely contribute to the retention of newly acquired PFP plasmids until they accumulate changes allowing their full compatibility with other resident plasmids. Indeed, we have shown that as little as five nt changes in the replication control region are sufficient to overcome incompatibility between PFP plasmid replicons [37].

The virulence plasmids pPsv48A and pPsv48C are structurally very fragile, experiencing high frequency intramolecular deletions and reorganizations promoted by the mobile genetic elements MITE*Psy2* and IS*801*. The TA systems carried by these plasmids, however, significantly reduce the accumulation of structural variants by selectively excluding them from the bacterial population. TA systems are bicistronic operons coding for a stable toxin and an unstable antitoxin that neutralises the activity of the toxin [51]. If the operon is lost, for instance due to a deletion, the antitoxin is rapidly degraded and bacterial growth is arrested due to the action of the stable toxin; thus, only cells that did not suffer the deletion and still contain the TA system can grow.

Our functional inactivation of the TA systems significantly increased the frequency of the pPsv48C deletions mediated by MITE*Psy2* by 50 times and by three times those mediated by IS*801*. This would indicate that the TA systems might be only moderately successful in preventing deletions mediated by IS*801*. However, we should consider that inactivation of the TA systems lead to a fivefold increase in the loss rate of gene *idi*, which is essential for tumour formation in the plant host [35]. Noticeably, it appears that the loss of gene *idi* was reduced even in those cases where deletion of this gene would not determine loss of any TA system (Fig. 5a). This could be a general feature, because a TA system from a virulence plasmid of *Shigella* spp. favoured the retention of nearby sequences, maintaining plasmid integrity [52].

Likewise, the occurrence of intramolecular deletions and reorganizations of pPsv48A increased three times upon functional inactivation of its TA systems (Table 3). This phenomenon has been termed post-recombinational killing [52], whereby the occurrence of insertion sequence-mediated rearrangements involving the deletion of TA systems lead to bacterial growth arrest and the consequent exclusion of the reorganized variants from the bacterial population. The modest protection offered by TA systems of pPsv48A is predictably an underestimate because of the limited number and types of events that we could detect with the pPsv48A::Tn*5*-GDYN1 construct used. Nevertheless, the TA systems of pPsv48A are contributing to the maintenance of virulence gene *ptz* (Table 3), which is essential for the induction of full-size tumours and the development of mature xylem vessels within them [18]. The occurrence of multiple, apparently redundant, TA systems in plasmids is intriguing. However, plasmids are highly dynamic entities undergoing a continuous trade of genetic material [2, 4]; as such it is feasible that multiple TA systems are selected to ensure the survival of different plasmid fragments. This is clearly exemplified by the 8.3 kb fragment that is “protected” by TA8 (Fig. 3).

In this work, we concentrated on examining the plasmids of strain NCPPB 3335. However, we would expect that the structural fragility of native plasmids and the protective role of TA systems are common phenomena in the *P. syringae* complex, and likely in other plant pathogens, for three main reasons. First, repetitive mobile genetic elements, and particularly IS*801*, are widespread in the *P. syringae* complex, can represent at least one third of diverse native plasmids, and are often associated to virulence genes [18, 19, 22, 27, 53]. IS*801* is remarkable, because it can efficiently transpose with a transposase provided *in trans* and because it follows a rolling circle replicative mechanism, leading to permanent insertions [19, 46, 47]. This implies that any fragment of IS*801* containing *ori801* is potentially mobilizable, that every transposition generates a potentially recombining site, and that one-ended transposition events can immediately lead to the generation of small to very large plasmid deletions. Additionally, other highly repetitive genes, such as the *rulAB* operon for resistance to UV light and many other DNA repair genes, are also commonly associated to virulence and other adaptive genes in *P. syringae* and many other bacteria [54–56]. All these repetitive genetic elements favour the mobility of virulence genes, promoting the high plasticity and adaptability of native plasmids [6, 16–18]; however, at the same time, represent recombination hotspots that can mediate deletion of key virulence genes [57], as highlighted by our results, and of many other adaptive genes. Second, the frequencies of recombination between MITEs and of transposition of IS*801* were very high, suggesting that they could be very active in promoting genomic changes. Third, and although largely ignored, TA systems are increasingly being found associated to native plasmids in many diverse plant pathogens, including *P. syringae* (see also Fig. 6) [17, 58, 59]. It is also noteworthy that most of these plasmids possess several TA systems, as occurs with plasmids from other bacteria [4, 57, 58].

## Conclusions

Here we show that TA systems are frequently found in plasmids of *P. syringae* and that they significantly contribute to plasmid stability, to preserve plasmid integrity and to maintain virulence genes in free living conditions. TA systems have been involved in a disparity of functions including, among others, the stabilization of plasmids and other mobile genetic elements, biofilm formation, modulation of bacterial persistence, resistance to antibacterial compounds, and prevention of large scale deletions in the chromosome, plasmids and episomes [51, 52, 60–62]. Our results show that genes found in plasmids of the plant pathogen *P. syringae* can be eliminated with high frequency because of plasmid loss and rearrangements mediated by mobile genetic elements. The occurrence of multiple toxin-antitoxin systems in plasmids effectively increase the survival of virulence genes and virulence plasmids in bacterial populations, facilitating their preservation in a diversity of environments lacking the strong selective pressure exerted by the plant host.

## Methods

### Bacterial strains, plasmids and growth conditions

Table 2 summarizes strains, native plasmids and constructions used in this study. LB medium [63] was routinely used for growing both *E. coli* (at 37 °C) and *Pseudomonas* strains (at 25 °C). Counter selection of cells carrying the *sacB* gene, which confers lethality in the presence of sucrose, was carried out in nutrient agar medium (Oxoid, Basingstoke, UK) supplemented with 5% sucrose (medium SNA). When necessary, media were supplemented with (final concentrations, in μg ml^− 1^): ampicillin, 100; gentamicin, 12.5; kanamycin, 7 for *P. syringae* and 50 for *E. coli*; tetracycline, 12.5.

### General molecular procedures and bioinformatics

DNA was amplified using a high fidelity enzyme (PrimeStar HS, Takara Bio Inc., Japan), or a standard enzyme (BIOTaq, Bioline, UK), and primers detailed in Additional file 1 Table S1. Amplicons were cloned using the CloneJET PCR Cloning Kit (Thermo Scientific) or the pGEM-T Easy Vector System (Promega). Purification of plasmids from *E. coli* was carried out following a boiling method [64] or using a commercial kit (Illustra plasmidPrep Mini Spin Kit, GE Healthcare). For plasmid profile gels, DNA was purified by alkaline lysis and separated by electrophoresis in 0.8% agarose gels with 1xTAE as described [25]. Plasmids were transferred to *P. syringae* by electroporation [65].

DNA sequences were compared and aligned using the BLAST algorithms [66], as well as the on-line MULTALIN [67] and EMBL-EBI server tools (http://www.ebi.ac.uk/Tools/msa/). The InterPro interface [68] (http://www.ebi.ac.uk/interpro/) was used to search for protein motifs. Nucleotide sequence visualization and manipulation was performed using the Artemis genome browser and ACT [69]. Primers were designed using the Primer3plus software [70].

### Manipulation of native plasmids of *P. syringae* pv. savastanoi

Native plasmids of *P. syringae* pv. *savastanoi* were tagged with Tn*5*-GDYN1 by conjugation using *E. coli* S17.1 as a donor; this transposon carries the levansucrase gene *sacB*, which allows for the identification of derivatives cured of plasmids by selection in medium with sucrose [18, 43]. Sites of Tn*5*-GDYN1 insertion were determined by sequencing of cloned EcoRI fragments containing the Gm^R^ end of the transposon and the adjacent sequences using primer IS50_F (Additional file 1 Table S1).

We constructed a derivative of pPsv48C containing a Km^R^-*sacB* cassette, immediately 5′ of the IS*801* isoform (100 nt upstream), as a tool to analyse the diverse deletions generated by the activity of this mobile element. The Km^R^-*sacB* cassette was amplified from pK18*mobsacB* [71] by PCR with specific primers (Additional file 1 Table S1), and introduced into an EcoRV site of pPsv48C (position 26,919 in accession no. FR820587) by allelic exchange recombination.

### Estimation of plasmid copy number

Plasmid copy number was estimated by quantitative PCR (qPCR) using as template total DNA purified with the JET flex Genomic DNA Purification Kit (Genomed, Germany). qPCR was performed using the CX96™ Real-Time System and analysed using CFX Manager software version 3.0 (BioRad, CA, USA), essentially as described [72] . A ten-fold serial dilution series of DNA was used to construct the standard curve for the single-copy chromosomal gene *gyrA*, used as reference [72], and the plasmids genes *ptz* (PSPSV_A0024; pPsv48A), *hopAO1* (PSPSV_B0010, pPsv48B) and *idi* (PSPSV_C0024, pPsv48C), using the primers indicated in Additional file 1 Table S1. Plasmid copy numbers were estimated using the ΔΔCt method [73, 74].

### Identification of putative plasmid stability determinants

For identification of putative stability determinants from plasmids pPsv48A (FR820585), pPsv48B (FR820586) and pPsv48C (FR820587), we manually inspected the annotation of the three plasmids and searched for those CDSs containing terms (stability, partition and related forms), or whose products contained typical domains associated to plasmid maintenance. Additionally, we selected putative toxin-antitoxin operons with a significant score (higher than 70) in the web tool RASTA-bacteria [75]. The complete set of loci identified and tested is summarized in Table 1.

The functionality of toxin genes from the putative TA systems was tested using the expression vector pBAD24 [76]. Toxin genes were amplified by high-fidelity PCR using primers with adapters for KpnI and PstI (Additional file 1 Table S1), cloned in the same sites of pBAD24, generating translational fusions with the first or second codon of the toxin gene, and transformed into *E. coli* NEB10β. Single colonies of appropriate clones grown overnight on LB + Amp were resuspended in LB, and two wells per clone of a microtiter plate were inoculated with 5 μl of the bacterial suspension and 150 μl of LB + Amp. Plates were incubated in a BioTek Gen5 (BioTek Instruments, VT, USA) microplate reader at 37 °C with 3 min of shaking every 15 min; after 3–4 h, one of the wells for each clone received 0.5% arabinose (final concentration, to induce the P_BAD_ promoter) and the other well received 0.2% glucose (final concentration, to further repress the P_BAD_ promoter). The OD_600_ of each well was recorded every 15 min, for a total of 20 h. The fidelity of clones was confirmed by sequencing, and at least four independent clones were tested for each toxin gene.

### Replication and stability assays

For functional analyses, the putative stability determinants from the three native plasmids of NCPPB 3335 (Table 1) were amplified by PCR with their own promoters, using specific primers, and cloned as BamHI fragments into the polylinker of vector pKMAG-C (construct 1 in Fig. 2). pKMAG-C replicates in *E. coli* through a p15a replicon and in pseudomonads through the cloned RepA-PFP replicon from pPsv48C [37]. The stability of these constructions, as well as that of the RepA-PFP and RepJ replicons from the pPsv48C plasmid and previously constructed chimeras [37], was tested after transformation into the plasmidless strain *P. syringae* pv. syringae B728a, essentially as described [77]. Briefly, transformants were grown overnight on LB plates with kanamycin, and twenty colonies per clone were collected and resuspended together in 500 μl of Ringer’s solution (1/4 strength; Oxoid, Basingstoke, UK). Serial dilutions were then plated on LB agar to get isolated colonies and, once developed, 100 colonies were picked to LB plates with and without kanamycin to determine the percentage of plasmid-containing colonies (Km^R^). The same procedure was followed to test these constructs in strain UPN912. The unstable cloning vector pKMAG-C was also included in the analyses as the baseline reference.

The stability of the minimal RepJ replicon [37], cloned into pKMAG (construct 4 in Fig. 2), was compared to that of plasmid pPsv48CΔ25, a naturally occurring 5.5 kb deletion derivate of pPsv48C that contains the RepJ replicon plus around 2 kb of downstream DNA, but no other maintenance systems. Both plasmids were maintained in strains derived from NCPPB 3335 and with no other native plasmids. Short-term stability was evaluated as stated above for strain B728a. For long-term stability, three independent LB cultures of each strain were started from single colonies and incubated at 25 °C with shaking and, after overnight growth, 10 μl of each culture were transferred to 3 ml of LB and incubated in the same conditions. We obtained LB plates containing 200–300 colonies both from the starting culture, immediately after single-colony inoculation, and after seven serial transfers in LB. These colonies were transferred to nylon membranes and analysed by colony hybridization [63], using an internal probe for *repJ*. The number of hybridizing colonies out of the total was scored to assess the prevalence of the RepJ replicon in both populations.

### Inactivation of TA systems

To evaluate the role of TA systems on plasmid maintenance, we proceeded to their functional inactivation, by supplying *in trans* the cognate antitoxins cloned in the broad-host range vector pRK415; resulting in the neutralization of the toxin by the cloned antitoxin, as described [44]. Antitoxin genes PSPSV_A0043, PSPSV_A0032 and PSPSV_A0020 from pPsv48A were amplified by PCR with their own promoters, cloned into pGEM-T Easy, excised as BamHI or NcoI-SacI (for PSPSV_A0032) fragments, and sequentially cloned into the BamHI, NcoI-SacI and BglII sites of vector pME6041, respectively. Primers A1_R and TA3_F were used to amplify these three elements as a single fragment, which was cloned into pJET 2.1 (CloneJET PCR Cloning Kit, Thermo Scientific), excised as a BglII fragment and cloned into the BamHI site of pRK415, downstream of the constitutive P_*lac*_ promoter in the vector, resulting in pRK3A. Essentially the same procedure was followed to clone in tandem and in this order, using primers A6_R and TA8_F, antitoxin genes PSPSV_C0050, PSPSV_C0008 and PSPSV_C0003 from pPsv48C into the vector pRK415, resulting in pRK3C. The integrity and fidelity of all clones was confirmed by nucleotide sequencing.

### Statistical procedures

All data are given as the mean ± standard deviation (sd). Each experiment was repeated from three to six times, with three technical replicates for each of the conditions tested. Means were compared using an analysis of variance (ANOVA) followed, when needed, by Duncan’s multiple range test (*p* < 0.05). We used software R Project 3.3.3 (R Core Team (2017); Vienna, Austria) to perform the statistics.

## Additional file

Additional file 1: **Table S1.** List and application of primers used in this work. (PDF 186 kb)

## Abbreviations

MITE: Miniature inverted-repeat transposable element
PFP: pPT23A-family plasmids
SD: Stability determinant
sd: Standard deviation
suc^R^: Resistant to 5% sucrose
TA: Toxin-antitoxin system
