# Supplementary material for: The toxic guardians — multiple toxin-antitoxin systems provide stability, avoid deletions and maintain virulence genes of Pseudomonas syringae virulence plasmids
Source: Mob DNA. 2019 Jan 31;10:7. doi: 10.1186/s13100-019-0149-4 (PMC6354349; doi:10.1186/s13100-019-0149-4)
Supplement: Supplementary file 1 — Table S1. List and application of primers used in this work. (PDF 186 kb) [file 13100_2019_149_MOESM1_ESM.pdf]

The toxic guardians — Multiple toxin-antitoxin systems provide stability, avoid deletions and maintain virulence genes of *Pseudomonas syringae* virulence plasmids

Leire Bardaji,[1] Maite Añorga,[1] Myriam Echeverría,[1] Cayo Ramos,[2] Jesús Murillo[1]*

Table S1 List and application of primers used in this work.

Primer name[a]	Sequence[b]	5´pos[c]	Remarks
**Cloning of putative stability determinants**			
**pPsv48A (accession no. FR820585)**			
SD1_F	GGATCCGATGGTAACCGCCAATGTTC	21649*	Cloning of SD1 into pKMAG-C
SD1_R	GGATCCTTCTGGGATTCCGGCTTAC	20277	
TA1_F	GGATCCACATAGGCTTGCGATTCAGC	24281*	Cloning of TA1 into pKMAG-C
TA1_R	GGATCCTTGATTGCTAATTCCGACACTG	23449	
TA2_F	GGATCCTTCCATCTCGTTTGCTGATG	37991*	Cloning of TA2 into pKMAG-C
TA2_R	GGATCCCGTTCCTCTATCGGTCTTCG	37061	
SD2_F	GGATCCATGCACTTCATCGGGTAGATTTC	53332*	Cloning of SD2 into pKMAG-C
SD2_R	GGATCCCATGAGCGAAGTGTAGTGCAG	52067	
TA3_F	GGATCCCCACGTAGTAGCCCGATGC	53072	Cloning of TA3 into pKMAG-C

TA3_R	GGATCCTCAGGGAGCACGAAACATC	54708*	
TA4_F	GGATCCGATCACCGCTGACATCGAG	64737*	Cloning of TA4 into pKMAG-C
TA4_R	GGATCCCGGCCAAGAACAAGAAAGAC	63811	
SD3_F	GGATCCACCCGTGTAGCGGTTAATTC	79421	Cloning of SD3 into pKMAG-C
SD3_R	GGATCCTTCCAAAAAGCCCGGTTAC	79884*	
**pPsv48B (accession no. FR820586)**			
TA5_F	GGATCCCTTGGATACGCACCGAAAAC	11832*	Cloning of TA5 into pKMAG-C
TA5_R	GGATCCAGGTGTCAATTGGCGATACC	11108	
SD4_F	GGATCCCCCAAATAGACCCAGCATATC	11884	Cloning of SD4 into pKMAG-C
SD4_R	GGATCCGGATGGTTACCGCCATACC	13274*	
SD5_F	GGATCCACCGTCGAAGCCAAGGTG	31991	Cloning of SD5 into pKMAG-C
SD5_R	GGATCCGCTGCCGCACTCGAAATAG	32672*	
SD6_F	GGATCCAGCGCACACTTGACGCTAC	35775	Cloning of SD6 into pKMAG-C
SD6_R	GGATCCCTATGACACCCGCTGACTTTC	37405*	
**pPsv48C (accession no. FR820587)**			
TA6_F	GGATCCCTGCCAAGAAAGTCGTCAAG	1760	Cloning of TA6 into pKMAG-C
TA6_R	GGATCCCGAAGCATGAATACCAGCAC	2442*	

TA7_F	GGATCCTTTTCGACCGGAATTGATTG	4752*	Cloning of TA7 into pKMAG-C
TA7_R	GGATCCGTACCCCATCATGCGATTG	3866	
SD7_F	GGATCCCTTCATGGAAAATGCTACCG	11121	Cloning of SD7 into pKMAG-C
SD7_R	GGATCCCCTTAATAGGTAGGTGTGAGCTATCC	9861*	
TA8_F	GGATCCGCAATACAGTGCAAACCAATG	39466	Cloning of TA8 into pKMAG-C
TA8_R	GGATCCTGATCAACATCCCCCAACAG	40245*	
**Regulated expression of toxin genes in E. coli**			
**pPsv48A**			
TA1_tox_F	TATGGTACCAGCAAAGAAGCCCGCAAGAC	23850*	Cloning of toxin from TA1 into pBAD24
TA1_tox_R	TATCTGCAGGCCATTCCACAGGCATAAAG	23496	
TA2_tox_F	TATGGTACCAGAATTGAAGTGGACAAGCAAG	37340*	Cloning of toxin from TA2 into pBAD24
TA2_tox_R	TATCTGCAGCGTTCCTCTATCGGTCTTCG	37061	
TA3_tox_F	TATGGTACCAATCCTGCTCGATACGAACG	53548	Cloning of toxin from TA3 into pBAD24
TA3_tox_R	TATCTGCAGCCTGATTCAACACGACGTTTAC	53982*	
TA4_tox_F	TATGGTACCCGTGCTAACAATTCGCTG	64278*	Cloning of toxin from TA4 into pBAD24
TA4_tox_R	TAACTGCAGACGGTTCAAGGGTATTGCTG	63995	
**pPsv48B**			

TA5_tox_F	TATGGTACCGATGCGAGGTGACCTAGTCA	11434*	Cloning of toxin from TA5 into pBAD24
TA5_tox_R	TATCTGCAGGCGACACAAGGAAAAGGTGT	11094	
**pPsv48C**			
TA6_tox_F	TATGGTACCACTACCCATTTTCTGGCTAG	2108	Cloning of toxin from TA6 into pBAD24
TA6_tox_R	TATCTGCAGCGAAGCATGAATACCAGCAC	2442*	
TA7_tox_R	TATGGTACCACAAGTAATCGTCACAGAAG	4269*	Cloning of toxin from TA7 into pBAD24
TA7_tox_F	TATCTGCAGGTCGATCTTCTCGGCCAGTC	3938	
TA8_tox_F	TATGGTACCACAAATCATCTGGCGGCAAC	39831	Cloning of toxin from TA8 into pBAD24
TA8_tox_R	TATCTGCAGATCAACATCCCCCAACAGC	40243*	
**Construction of pRK3A and pRK3C**			
**pPsv48A**			
A1_R	GGATCCATCCCTGGCTTCGGTCATAG	23716	with TA1_F
A2_R	GGATCCGGCCAGAAACTCGTATAGCC	37281	with TA2_F
A3_R	GGATCCGCTGCGGTTCTGAAATTACG	53584*	with TA3_F
**pPsv48C**			
A6_R	GGATCCGTTCTGAAAGGGGCAGAACG	2240*	with TA6_F

A7_R	GGATCCAGCTTCTGGGTCCTTTGCAG	4189*	with TA7_F
A8_R	GGATCCGCTTGCGGGTCTTCGTTG	39907*	with TA8_F
**PCR multiplex**			
**pPsv48C**			
RepL_F	TACGCTACGCGAGCTCAATC	33336	Detection of PSPSV_C0043
RepL_R	TCTAGAGGCCTTCCTTATTCATGATGC	34355*	
7C_F	AAACAGCCCCCGACATTG	4142*	Detection of PSPSV_C0007
7C_R	GCCGAAATTTCCGTCAGG	3411	
RepJ_F	ATAGGCTCCTAGCGTCGAG	30484	Amplifies a region within RepJ replicon
RepJ_R	AGAGCGATTGACCATGCAC	31028*	
RepA_F	CAATAGCAGCTTGTCGCTCA	882	*repA* detection
RepA_R	GTTACCGTTGGCCTCTCGTC	1148*	
ipt_F	GCGTCTCCAGGAAGAATTGG	18715	To detect gene *idi* and estimate copy number
ipt_R	ACCTCATCCGGATTCAAGC	18870*	
**Plasmid copy number estimation by qPCR**			
**Chromosome (accession nº CP008742)**			
gyrA_F	GACGAGCTGAAGCAGTCCTACC	3774872*	Internal to *gyrA*

gyrA_R	TTCCAGTCGTTACCCAGCTCG	3774736	
**pPsv48A**			
ptz_F	AAGACCCATACTGGTGCGATAG	28623*	Internal to *ptz*
ptz_R	CCGCAACCAACTCGTCTAAC	28471	
**pPsv48B**			
AO1_F	AGGGAAACCGAAGACCAATG	10026	Internal to *hopAO1*
AO1_R	AACCAGCCAATTTCAGATCG	10176*	
**pPsv48C**			
ipt_F	GCGTCTCCAGGAAGAATTGG	18715	To detect gene *idi* and estimate copy number
ipt_R	ACCTCATCCGGATTCAAGC	18870*	
**Other purposes**			
pk18mob-km-sac_F	GATATCTTATGGACAGCAAGCGAACC		Used to amplify Km^R-*sacB* cassette from pK18*mobsacB*
pk18mob-km-sac_R	GATATCTCGTGGACTATGAGCACGTC		
IS801_R	ACGCATCAGCGTCTTCCTAC		Anneals 74 nt upstream of the 3´ end of CRR1; outwards
IS50_F	ACACAGATTTAGCCCAGTCG		Anneals in Tn*5*GDYN1

[a] The name of the primer is indicated, as well as the molecule used as target DNA and the purpose of designing each pair.

[b] Adaptors introduced in primers to facilitate cloning are underlined.

[c] The coordinates of the annealing point of the first nucleotide of each primer is indicated. Asterisks indicate that the primer anneals in the reverse strand of the DNA molecule.
